# Effectiveness of SARS‐CoV‐2 testing strategies: A scoping review

**DOI:** 10.1002/cesm.12030

**Published:** 2023-11-21

**Authors:** KM Saif‐Ur‐Rahman, Ani Movsisyan, Kavita Kothari, Thomas Conway, Marie Tierney, Caoimhe Madden, Petek Eylul Taneri, Jane A. O'Halloran, Nadra Nurdin, Lena Murphy, Deirdre Mulholland, Andrea C. Tricco, Declan Devane

**Affiliations:** ^1^ Evidence Synthesis Ireland and Cochrane Ireland University of Galway Galway Ireland; ^2^ School of Nursing and Midwifery University of Galway Galway Ireland; ^3^ Consultant to Methods and Standards Team World Health Organization Geneva Switzerland; ^4^ Consultant to Library & Digital Information Networks World Health Organization Kobe Japan; ^5^ HRB‐Trials Methodology Research Network University of Galway Galway Ireland; ^6^ School of Nursing, Psychotherapy and Community Health Dublin City University Dublin Ireland; ^7^ UCD Centre for Experimental Pathogen Host Research (CEPHR) University College Dublin Dublin Ireland; ^8^ Royal College of Physicians Dublin Ireland; ^9^ Occupational Health Healthy Workplace Unit Dublin North Ireland; ^10^ Public Health Health Service Executive Dublin Ireland; ^11^ Li Ka Shing Knowledge Institute, St. Michael's Hospital Unity Health Toronto Toronto Ontario Canada; ^12^ Epidemiology Division and Institute of Health Policy, Management, and Evaluation, Dalla Lana School of Public Health University of Toronto Toronto Ontario Canada; ^13^ Queen's Collaboration for Health Care Quality: A JBI Centre of Excellence Queen's University Kingston Ontario Canada

**Keywords:** COVID‐19, SARS‐CoV‐2, scoping review, testing strategy

## Abstract

**Introduction:**

Rapid identification of severe acute respiratory syndrome‐coronavirus 2 (SARS‐CoV‐2) infections by testing potentially reduced coronavirus disease‐19 (COVID‐19) cases. Testing strategies varied across countries and during different stages of the pandemic. This scoping review aims to map the available evidence on the effectiveness of SARS‐CoV‐2 testing strategies for suspected cases and asymptomatic populations to inform the development of World Health Organization recommendations for SARS‐CoV‐2 testing strategies.

**Methods:**

We followed the standard methods for scoping reviews. We searched Medline (OVID), EMBASE (Elsevier), and Europe PMC using a comprehensive search strategy. The search was conducted in January 2023 and covered the period from January 2020 to January 2023. Two review authors independently screened the titles and abstracts, and full texts. Data were extracted onto a pilot‐tested form by a review author and cross‐checked by another review author. We provided a descriptive report summarizing the extracted data around the outcomes and created an interactive map of the available evidence using the evidence for policy and practice mapper.

**Results:**

We identified 34,550 citations from the databases. After the screening, we included 17 studies from 11 countries for data extraction. The study designs were randomized controlled trials (*n* = 3), nonrandomized experimental studies (*n* = 3), cohort studies (*n* = 3), cross‐sectional studies (*n* = 4), self‐controlled case series (*n* = 1), and economic evaluations (*n* = 3).  Among the included studies, 14 used reverse transcription‐polymerase chain reaction and 10 studies used antigen‐detecting rapid diagnostic test. The settings of the studies were healthcare facilities (*n* = 8), communities (*n* = 4), schools, and workplaces (*n* = 3). Included studies considered symptomatic and asymptomatic individuals, or both, or asymptomatic contacts. Most of the studies (*n* = 14) reported the COVID‐19 positivity rate as the primary outcome. Other reported outcomes are the number of COVID‐19 cases (*n* = 11), number of hospitalizations and deaths (*n* = 3), and cost (*n* = 3).

**Conclusion:**

We identified evidence gaps in the effectiveness of SARS‐CoV‐2 testing strategies, particularly in specific settings such as schools and long‐term care facilities. This scoping review provides a foundation for further research, allowing researchers and stakeholders to focus on addressing the identified gaps.

## 1 INTRODUCTION

Severe acute respiratory syndrome‐coronavirus 2 (SARS‐CoV‐2), the etiological agent causing coronavirus disease‐19 (COVID‐19), emerged in December 2019 and spread rapidly around the world [1]. The COVID‐19 pandemic has resulted in severe threats to global health, unprecedented challenges to health systems, and widespread disruption of daily life [2]. It has impacted the economy, public health, and mental health [3]. As of April 2023, 764 million confirmed cases of COVID‐19, including 6.9 million deaths, have been reported to the World Health Organization (WHO) [4]. The spread of SARS‐CoV‐2 primarily occurs through respiratory droplets and aerosols that are expelled during coughing and sneezing. However, the rapid and exponential transmission of the virus can be partially attributable to individuals who are presymptomatic or asymptomatic transmitters [5, 6].

Globally, government responses to the pandemic have varied widely. Containment strategies include stay‐at‐home requirements, school, workplace, public transport closures, gathering size restrictions, internal movement restrictions, and international travel. Health systems strategies include testing policy, contact tracing, investment in health care, and vaccination [7]. The WHO endorses several of these public health and social strategies, including surveillance and response, in which testing is a critical component [8]. Identifying a positive case through testing enables contact tracing, isolation, quarantine, and genetic sequencing, which are crucial measures to control the transmission of SARS‐CoV‐2 [9]. In addition, testing strategies have been proposed to reverse containment measures such as school and workplace closures and travel restrictions [10, 11]. Significant investments in large‐scale testing initiatives have been implemented worldwide since the onset of the pandemic [12], with upscaling of laboratory infrastructure being an integral component of the public health response. Testing strategies vary across countries and during different stages of the pandemic, ranging from targeting suspected cases based on symptoms or epidemiological criteria to widespread screening of asymptomatic populations [13].

Testing methods for diagnosis of SARS‐CoV‐2 infection include nucleic acid amplification tests (NAAT), including reverse transcription‐polymerase chain reaction (RT‐PCR), which has been described as the gold standard for testing with high sensitivity and specificity [14]. The early stages of the pandemic were characterized by shortages in RT‐PCR kits in many parts of the world, leading to a lack of access to testing or testing only in limited circumstances [15]. Although lower in diagnostic accuracy, antigen‐detecting rapid diagnostic test (RDT) has gained acceptance due to the potential to increase access to testing, rapid detection of infections [16], and the reduced cost and labor associated with antigen‐detecting RDT administration. Both RT‐PCR and antigen‐detecting RDT can be performed using a variety of specimens, including nasopharyngeal swabs, nasal swabs, sputum, and saliva samples [17].

Rapid identification of SARS‐CoV‐2 infections can potentially reduce COVID‐19 cases through timely quarantine and contact tracing. A rapid review by Walsh et al., published in September 2022, has explored the effectiveness of antigen‐detecting RDT in reducing transmission among asymptomatic individuals [18]. This review could not demonstrate the effectiveness of antigen‐detecting RDT amongst asymptomatic individuals to reduce transmission of SARS‐CoV‐2 due to inconsistent results and a lack of evidence available at the time of data screening. Mathematical modelling studies suggest large‐scale testing may be a cost‐effective method to limit community transmission of SARS‐CoV‐2 [19]. However, there is limited knowledge and “real‐world” evidence of the impact of specific SARS‐CoV2 testing strategies on limiting community transmission, morbidity, mortality, and the costs associated with testing strategies.

This scoping review aims to map the available evidence on the effectiveness of SARS‐CoV‐2 testing strategies for suspected cases and asymptomatic populations. The scoping review was commissioned by WHO and will inform the development of recommendations for SARS‐CoV‐2 testing strategies to mitigate community transmission of SARS‐CoV‐2, as well as reduce morbidity and mortality in a cost‐efficient manner.

### 1.1 Review questions

1. What is the evidence on the effectiveness of testing (NAAT, such as PCR or antigen‐detecting RDT) suspected cases (including those at high risk) presenting to health services to limit community transmission of SARS‐CoV‐2, morbidity, mortality, and health care costs?
2. What is the available evidence on the effectiveness of testing (NAAT, such as PCR or antigen‐detecting RDT) of asymptomatic populations (including contacts and those at high risk of exposure, such as health workers) to limit community transmission of SARS‐CoV‐2, morbidity, mortality, and health care costs?

This scoping review of the effectiveness of SARS‐CoV‐2 testing strategies will be an important resource for researchers and policymakers by providing an overview of the currently available evidence in SARS‐CoV‐2 testing strategies. Its principal objective is to systematically collate and present the existing studies and findings in the field rather than analyze the effectiveness of various testing methods. It sets the stage for evidence‐based decisions about the need and feasibility of additional primary research or more refined evidence synthesis. Based on the evidence map from this review, future investigations could focus on specific testing strategies, settings, or outcomes.

## 2 METHODS

### 2.1 Protocol and registration

The scoping review protocol followed the JBI (formerly Joanna Briggs Institute) guide to scoping reviews [20, 21], and the final review was reported by the Preferred Reporting Items for Systematic Review and Meta‐analysis Extension for Scoping Reviews (PRISMA‐ScR) [22]. The protocol was registered in the Open Science Framework (https://osf.io/).

### 2.2 Eligibility criteria

#### 2.2.1 Population

Eligible participants included members of the general population (adults and children) who either:

(a) met the WHO suspected case definition of SARS‐CoV‐2 infection, which is described as “a person who meets the clinical or epidemiological criteria, a patient with severe acute respiratory illness, a person with no clinical signs or symptoms or meeting epidemiologic criteria with a positive professional‐use or self‐test SARS‐CoV‐2 Antigen‐detecting RDT” [23]; or
(b) had no symptoms and may or may not have had known exposure to SARS‐CoV‐2 (e.g., contacts).

Data were disaggregated and tabulated if available for high‐risk symptomatic suspected cases and asymptomatic contacts. The included papers were required to define the population and state the criteria for testing.

#### 2.2.2 Intervention

Relevant interventions comprised test‐based screening strategies (including one‐off and routine testing) in any setting via antigen‐detecting RDT or NAAT, including RT‐PCR tests. The focus of this review is not on the diagnostic test accuracy of different tests.

#### 2.2.3 Comparator

‐ Testing strategy versus no testing.
‐ Testing strategy versus another testing strategy (e.g., antigen‐detecting RDT vs. NAAT testing, home‐based vs. health professional‐based, one‐off testing vs. routine testing, one frequency of testing vs. another frequency of testing, etc.).

#### 2.2.4 Outcomes

The review included the following categories of outcomes:

‐ COVID‐19 cases avoided due to the testing strategy.
‐ COVID‐19 positivity rate due to the testing strategy.
‐ COVID‐19‐related hospitalizations avoided due to the testing strategy.
‐ COVID‐19‐related deaths avoided due to the testing strategy.
‐ Costs associated with testing strategies.

All specific outcomes within these categories were considered (e.g., number of infections or probability of epidemic in the community as part of the category “cases avoided in the community”).

#### 2.2.5 Setting

As detailed above, we included all settings if the study describes a relevant population.

#### 2.2.6 Type of studies

We included the following types of studies:

1. Experimental and quasi‐experimental studies:
  ▪ Randomized trials, nonrandomized trials, controlled before–after studies, and interrupted time series.
  ▪ Instrumental variable and regression discontinuity studies as per Reeves et al. [24].

2. Observational studies:
  ▪ Studies that, through design, analysis, or both, allow for controlling only based on observed covariates. Such studies are more prone to selection bias. Study designs within this group include cross‐sectional studies, cohort studies (retrospective, nonconcurrent, and prospective), and case–control studies (retrospective and prospective).

3. Mathematical modelling studies. (The list of such studies was provided to the review commissioner. Mathematical modelling studies were not considered in the synthesis/mapping.)
  Studies from groups 1 and 2 above were required to meet at least one of the following criteria:
  - have collected quantitative data on at least one outcome within this review and at least once postintervention, or
  - have estimated the change over time in the effects of the intervention through the same or different individuals at multiple time points before and after the intervention, or
  - have estimated the differences between the groups receiving the intervention of interest or comparator.

#### 2.2.7 Type of publications

English language papers available in full text and published in peer‐reviewed journals or on preprint servers were included in the review. The review considered studies published from January 2020 onwards to capture the evidence on SARS‐CoV‐2 variants of concern.

#### 2.2.8 Exclusion criteria

The following types of studies were excluded from the review:

- Animal and in vitro studies.
- Diagnostic accuracy studies focused on test performance. These studies explore the ability of a diagnostic test to differentiate between healthy people and people with a health condition.
- Studies that did not provide a quantitative measure of effectiveness/impact (e.g., qualitative studies).
- Opinion pieces, editorials.
- Conference abstracts and reports.
- Systematic reviews and other evidence reviews.

### 2.3 Search methods for identification

#### 2.3.1 Electronic database

The search strategy was developed and executed by an information specialist (K. K.) with the assistance of topic and methods experts on the team. The search was conducted in January 2023 and covered the period from January 2020 to January 2023. We searched Medline (OVID), EMBASE (Elsevier), and Europe PMC. The comprehensive search strategy for all the databases can be found in Supporting Information S1: Tables 1–3.

### 2.4 Data collection and analysis

#### 2.4.1 Software

We deduplicated citations in Deduklick, an AI‐based software [25]. We screened citations and full texts in Covidence [26], a web‐based collaboration software platform specifically designed to streamline the production of systematic reviews and other types of evidence synthesis.

#### 2.4.2 Screening

The screening of citations was carried out in two phases, both of which underwent pilot testing. After deduplication, two reviewers independently screened the titles and abstracts of the citations for eligibility. Where disagreements occurred, a discussion involving a third reviewer was conducted to resolve the conflicts. Two independent reviewers screened full‐text articles, and any discrepancies were resolved through discussion with a third review author. Reasons for exclusion were documented during the full‐text screening phase.

#### 2.4.3 Data extraction

One reviewer extracted the data onto a pilot‐tested form. The extracted data included publication year, author name, types of study, sample size, study population characteristics (age, sex, ethnicity, and geographic location), symptomatic status, types of intervention/testing strategies, comparator, settings, and outcomes. We did not extract data from mathematical modelling studies. Another review author cross‐checked the extracted data to ensure accuracy.

The evidence was tabulated for the following dimensions:

Country/geographic region

Population

- Symptomatic individuals.
  ∘ High‐risk symptomatic individuals (i.e., known comorbidities, e.g., age, obesity, immunocompromise).
- Asymptomatic individuals with no known exposure.
- Asymptomatic contacts.
- Adults versus children.
- Health workers.
- Vaccinated versus unvaccinated individuals.

Eligible participants included members of the general population, both adults and children, who met either of the following criteria:

(a) Symptomatic individuals with symptoms of SARS‐CoV‐2.
(b) Susceptible individuals without any symptoms but at risk of contracting SARS‐CoV‐2.

The testing methods/techniques included in the review were:

- Test‐based screening strategies using antigen‐detecting RDT.
- Test‐based screening strategies using NAAT, including RT‐PCR.

The review considered the following testing approaches:

- One‐off testing: Testing conducted as a single occurrence.
- Routine testing: Regular and scheduled testing performed at predetermined intervals.
- Intermittent testing: Testing carried out periodically but not on a continuous or regular basis.

The review considered the following settings:

- School/academic institutions.
- Community.
- Long‐term residential care facilities.
- Healthcare facilities.
- Other settings (not specified).

The review categorized outcomes into the following categories:

- Cases avoided: Measuring the number of COVID‐19 cases that were prevented or avoided due to the testing strategy.
- SARS‐CoV‐2 positivity rate: Assessing the rate or percentage of positive SARS‐CoV‐2 cases detected through the testing strategy.
- Hospitalizations: Examining the impact of the testing strategy on preventing or reducing COVID‐19‐related hospitalizations.
- Deaths: Investigating the effect of the testing strategy on preventing or reducing COVID‐19‐related deaths.
- Costs: Evaluating the economic costs associated with the testing strategies implemented.

The review considered the following types of evidence:

- Randomized trials.
- Nonrandomized trials.
- Controlled before–after studies.
- Interrupted time‐series studies.
- Observational studies, including:
  ∘ Cross‐sectional studies.
  ∘ Cohort studies.
  ∘ Case–control studies.
- Mathematical modelling studies. (The list of such studies was provided to the review commissioner.)

We also considered the PRO‐EDI (tools to help reviewers make equity, diversity, and inclusion assessments) criteria to address equity issues during data extraction. The PRO‐EDI framework includes factors such as age, sex, gender, ethnicity, socioeconomic status, level of education, location, and other relevant factors that pertain to the review's focus on equity. This tool aims to highlight the gaps in evidence through the lens of equity, diversity, and inclusion.

#### 2.4.4 Risk of bias assessment

We did not assess the risk of bias of the studies included in this scoping review, as it is not typically considered relevant in scoping reviews [21, 27].

#### 2.4.5 Data synthesis

Our aim was not to synthesize the results but to map and summarize the available evidence. We intended to map and summarize the extent and type of the identified evidence rather than providing the actual results of individual studies. We provided a descriptive report summarizing the extracted data around the outcomes mentioned above for the populations studied (adults, children, symptomatic cases, asymptomatic individuals, etc.), as well as the testing strategies and the type of evidence. We provided descriptive frequencies and number of articles in terms of population, geographic region, testing strategy (RT‐PCR, or antigen‐detecting RDT), types of evidence (study design), settings (community, school, hospital, long‐term care facilities, etc.) and outcomes (cases, hospitalization, deaths, and cost). We mapped the data according to the test strategy and the outcomes. We mapped the countries and geographic regions where the studies were conducted. We also provided visual maps/graphs of the outcomes considering the population, study settings, and study types (where data are available) [28]. We provided a list of relevant mathematical modelling studies but did not include them in the evidence mapping.

## 3 RESULTS

### 3.1 Results of the search

**Figure 1:**
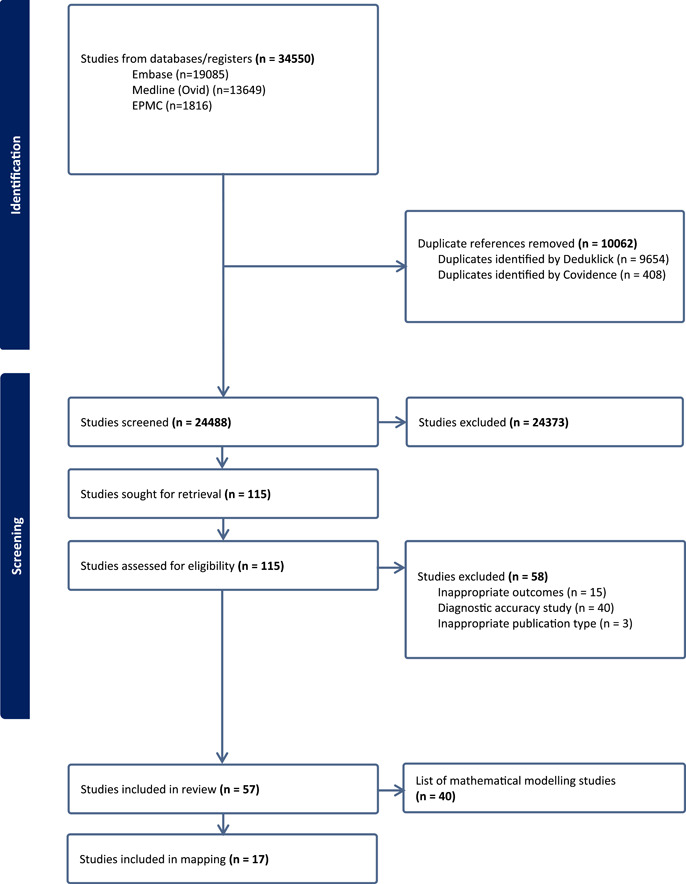
PRISMA‐ScR (Preferred Reporting Items for Systematic Review and Meta‐analysis Extension for Scoping Reviews) flow diagram. The bold values are the main values to visualize.

The PRISMA flow diagram presents the study selection process (Figure 1). We identified 34,550 citations from the databases, of which 10,062 duplicates were removed. Subsequently, we screened 24,488 citations in the title and abstract screening phase and excluded 24,373 that did not meet the eligibility criteria. We then proceeded to screen the remaining 115 citations in full text. At this stage, we excluded 58 articles for the following reasons: diagnostic test accuracy study (*n* = 40), inappropriate outcome (*n* = 15), and inappropriate publication type (*n* = 3). The complete list of excluded papers with the reasons for exclusion is provided in Supporting Information S1: Table 4.

Out of the included studies, 40 were mathematical modelling studies. However, according to the methods, mathematical modelling studies were not intended to be included in the mapping. The list of such studies was provided to the review commissioner. Therefore, 17 studies were considered for data extraction. The complete list of the mathematical modelling studies is provided in Supporting Information S1: Table 5.

### 3.2 Characteristics of the included studies

**Figure 2:**
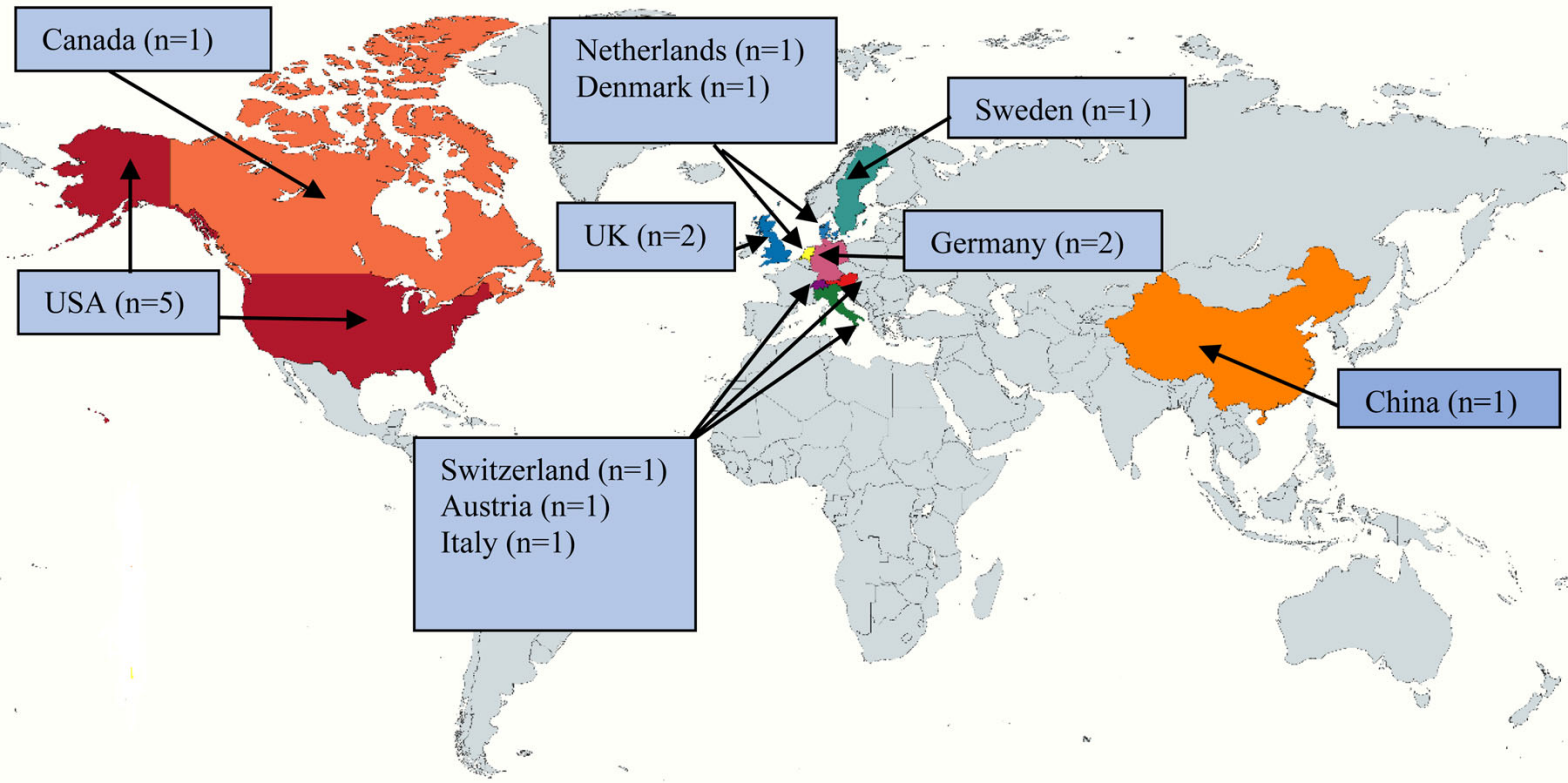
Geographic distribution of the included studies (*n* denotes the number of studies).

Among the 17 included studies, six were published in 2021 [29, 30, 31, 32, 33, 34], and the remaining 11 studies [35, 36, 37, 38, 39, 40, 41, 42, 43, 44, 45] were published in 2022. The study design of the included studies were randomized controlled trials [32, 34, 41], nonrandomized experimental studies [31, 33, 36], cohort studies [35, 42, 43], cross‐sectional studies [38, 39, 40, 44], self‐controlled case series (SCCS) [30], and economic evaluations [29, 37, 45]. Among the randomized controlled trials, two were cluster randomized controlled trials [32, 34]. The studies were conducted in 11 different countries. The geographic distribution of the included studies is presented in Figure 2. The sample size of the included studies ranged from 22 participants) [30] to 238,579 [32]. Detailed characteristics of the included studies are provided in Table 1.

**Table 1:** Characteristics of included studies.

Reference	Study design	Adult/children	Sex	Country	Sample size	Symptomatic/asymptomatic individuals	Vaccinated/Unvaccinated	Settings	Outcome reported
[41]	Randomized controlled trial	Adult	Male and female	UK	49,623	Asymptomatic contacts	Vaccinated and unvaccinated	Community	SARS‐CoV‐2 positivity rate; cases
[32]	Cluster randomized controlled trial	Adult and children	Male and female	UK	238,579	Asymptomatic contacts	Not reported	School; workplace	SARS‐CoV‐2 positivity rate; cases
[34]	Cluster randomized controlled trial	Adult and children	Male and female	Germany	3970	Asymptomatic individuals	Not reported	School; workplace	SARS‐CoV‐2 positivity rate; cases
[36]	Nonrandomized experimental study	Adult	Male and female	USA	8879	Symptomatic individuals	Not reported	Healthcare facilities	SARS‐CoV‐2 positivity rate; cases; hospitalization; ICU admission rate; death
[31]	Nonrandomized experimental study	Adult and children	Male and female	USA	128	Both symptomatic and asymptomatic individuals	Not reported	Community	SARS‐CoV‐2 positivity rate
[33]	Nonrandomized experimental study	Children	Male and female	USA	774	Symptomatic and asymptomatic individuals; asymptomatic contacts	Not reported	Community	Cases
[35]	Cohort study	Adult	Male and female	Sweden	2940	Symptomatic individuals	Not reported	Healthcare facilities	SARS‐CoV‐2 positivity rate; cases; hospitalization; ICU admission rate; death
[42]	Cohort study	Adult and children	Not reported	Canada	2385	Symptomatic and asymptomatic individuals; asymptomatic contacts	Vaccinated and unvaccinated	School; workplace	SARS‐CoV‐2 positivity rate
[43]	Cohort study	Adult and children	Male and female	Austria	Not reported	Symptomatic individuals	Not reported	Healthcare facilities	SARS‐CoV‐2 positivity rate; hospitalization; death
[38]	Cross‐sectional study	Adult (healthcare workers)	Not reported	USA	Not reported	Both symptomatic and asymptomatic individuals	Not reported	Healthcare facilities; community	SARS‐CoV‐2 positivity rate
[39]	Cross‐sectional study	Adult and children	Male and female	Netherlands	19773	Symptomatic and asymptomatic individuals; asymptomatic contacts	Not reported	Community	SARS‐CoV‐2 positivity rate
[40]	Cross‐sectional study	Children	Male and female	USA	197	Symptomatic individuals	Not reported	Healthcare facilities	SARS‐CoV‐2 positivity rate; cases
[44]	Cross‐sectional study	Children	Not reported	Italy	1313	Symptomatic individuals; asymptomatic contacts	Not reported	Healthcare facilities	SARS‐CoV‐2 positivity rate; cases
[30]	SCCS study	Adult	Male and female	China	22	Not reported	Not reported	Healthcare facilities	SARS‐CoV‐2 positivity rate; cases
[37]	Economic evaluation	Not reported	Not reported	Switzerland	128,842	Not reported	Not reported	Not reported	Cost
[45]	Economic evaluation	Not reported	Not reported	Germany	Not reported	Asymptomatic individuals	Not reported	Healthcare facilities	Cost
[29]	Economic evaluation	Adult	Male and female	Denmark	4811	Both symptomatic and asymptomatic individuals	Not reported	Healthcare facilities	SARS‐CoV‐2 positivity rate; cases; cost

Abbreviations: ICU, intensive care unit; SARS‐CoV‐2, severe acute respiratory syndrome‐coronavirus 2; SCCS, self‐controlled case series; USA, United States of America.

Six of the studies considered symptomatic and asymptomatic individuals [29, 31, 33, 38, 39, 42], of which three studies also considered asymptomatic contacts [33, 39, 42]. Two studies focused on asymptomatic contacts only [32, 41], four studies focused on symptomatic individuals only [35, 36, 40, 43], one study focused on both symptomatic individuals and asymptomatic contacts [44], two studies focused on asymptomatic individuals only [34, 45], and two studies did not specify symptom status [30, 37]. Only one study specifically mentioned healthcare workers as participants, in addition to the general community [38].

Two studies reported the vaccination status of participants [41, 42]. These studies considered both vaccinated and unvaccinated individuals.

### 3.3 Settings

Most studies (*n* = 8) were conducted in healthcare facilities [29, 30, 35, 36, 40, 43, 44, 45]. Four studies were conducted in the community [31, 33, 39, 41]. One study was conducted in a healthcare facility and the community [38]. Three studies were conducted in schools and workplaces (specifically for the school staff) [32, 34, 42]. One study did not specify the setting [37]. Among the studies conducted in school settings, only one study mentioned the inclusion of higher secondary classes (up to 21 years of age) [34].

### 3.4 Equity, diversity, and inclusion assessments

Six of the included studies were conducted with an adult population only [29, 30, 35, 36, 38, 41]. Three studies focused on children only [33, 40, 44]. Six studies considered both adults and children [31, 32, 34, 39, 42, 43], of which four reported the outcomes for children and adults separately [34, 39, 42, 43]. Two studies did not specify the age of the participants [37, 45]. Most of the studies included both males and females, although information on the sex of participants was not provided in five studies [37, 38, 42, 44, 45]. Ethnicity was specified in seven studies [32, 33, 36, 38, 39, 40, 41], while socioeconomic status was mentioned in only one study [41]. The education level attained by individuals was reported in three studies conducted in school settings, focusing on school students [32, 34, 42]. However, information on school staff's education level was not provided. Whether the study was performed in a rural or urban location was only reported in three studies [35, 39, 44], all performed in urban settings.

### 3.5 Testing method and frequency

Among the included studies, 14 used RT‐PCR [29, 30, 31, 33, 34, 35, 36, 37, 38, 39, 40, 41, 42, 44] and 10 studies used antigen‐detecting RDT [29, 32, 33, 35, 39, 41, 42, 43, 44, 45]. None of the studies mentioned using test‐based screening strategies using NAAT other than RT‐PCR.

Most of the studies comprised one‐off testing [29, 30, 31, 33, 35, 36, 38, 39, 40, 41, 42, 43, 44, 45], indicating that testing was conducted only once for each participant. Four studies mentioned routine testing [32, 34, 41, 42], which implies regular and repeated testing of individuals. Most routine testing studies were conducted in school settings, except for one shown in the community [41]. Only one study mentioned intermittent testing, which involved testing contacts exposed to a positive individual at the time of diagnosis in addition to routine testing [42]. Five studies specifically mentioned that the testing was free [29, 32, 34, 39, 40], indicating that participants did not have to bear any costs for the testing services.

### 3.6 Outcome categories

The outcomes reported by the included studies were as follows: COVID‐19 positivity rate, number of cases, hospitalization, intensive care unit (ICU) admission rate, death, and cost of testing.

Most studies (*n* = 14) focused on reporting the COVID‐19 positivity rate as the primary outcome [29, 30, 31, 32, 34, 35, 36, 38, 39, 40, 41, 42, 43, 44]. This outcome indicates the proportion of individuals who tested positive for SARS‐CoV‐2 among those who underwent testing.

Eleven studies reported the number of COVID‐19 cases as an outcome [29, 30, 32, 33, 34, 35, 36, 40, 41, 42, 44]. This outcome provides information on the total number of confirmed COVID‐19 cases identified through testing.

Three studies examined the number of hospitalizations and deaths as outcomes [35, 36, 43]. Among these three studies, two also reported the rate of ICU admission [35, 36], which indicates the proportion of COVID‐19 cases requiring ICU care.

In addition, three economic evaluation studies considered cost as an outcome [29, 37, 45]. Two of these studies solely reported costs [37, 45], while the remaining study included the COVID‐19 positivity rate, number of cases, and the cost of testing [29].

The characteristics of the included studies, categorized by the testing strategy, are presented in Table 2. This table provides a comprehensive overview of the studies, including details on the study design, population, settings, outcomes, and other relevant information as per different testing strategies (test‐based screening strategies using antigen‐detecting RDT and test‐based screening strategies using RT‐PCR).

**Table 2:** Characteristics as per testing technique.

	Test‐based screening strategies using antigen‐detecting RDT (*n*)	Test‐based screening strategies using RT‐PCR (*n*)
Study population characteristics		
Symptomatic individuals	7	10
High‐risk symptomatic individuals (i.e., known comorbidities, for example, age, obesity, immunocompromised)	1	1
Asymptomatic individuals with no known exposure	5	7
Asymptomatic contacts	6	5
Adult	7	9
Children	6	7
Health workers	0	1
Vaccinated	0	0
Unvaccinated	0	0
Both vaccinated and unvaccinated	2	2
Equality, diversity, and inclusion assessments (PRO‐EDI)		
Blinding (masking) mentioned	0	0
Randomization (How participants were allocated to interventions) mentioned	2	2
Ethnicity mentioned	4	6
Socioeconomic status mentioned	1	1
Level of education mentioned	2	2
Location—Urban	3	3
Location—Rural	0	0
Settings^a^		
School (<18 year olds)	2	2
College/university (>18 years old)	0	1
Long‐term residential care facilities	0	0
Healthcare facilities	5	7
Community	3	5
Workplace	2	2
Testing frequency		
One‐off testing	9	12
Routine testing	3	3
Intermittent testing	1	1
Place of testing^a^		
Clinic based/healthcare facility	4	8
Community based	3	5
Outcomes categories^a^		
COVID‐19 positivity rate	8	12
Cases	7	10
Hospitalizations	2	2
ICU admission rate	1	2
Deaths	2	2
Cost	2	2

Abbreviations: COVID‐19, coronavirus disease‐19; ICU, intensive care unit; RDT, rapid diagnostic test; RT‐PCR, reverse transcription‐polymerase chain reaction.

^a^ Both antigen‐detecting RDTs and RT‐PCR were used in some studies.

We have described the different testing methods and corresponding outcomes in terms of SARS‐CoV‐2 positivity rate, number of cases, hospitalization rate, ICU admission rate, death rate, and cost in Table 3.

**Table 3:** Outcome measurement of different testing methods.

Reference	Testing method	Comparator (alternative testing methods)	Outcome measurements
[41]	RT‐PCR	Innova LFD tests (Innova SARS‐CoV‐2 antigen‐detecting RDT)	SARS‐CoV‐2 positivity rate: ‐ RT‐PCR 8.9% ‐ Antigen‐detecting RDT 8.9% Cases: ‐ RT‐PCR 2364 ‐ Antigen‐detecting RDT 2330
[32]	Antigen LFDs	No testing	SARS‐CoV‐2 positivity rate: Rate of symptomatic PCR‐confirmed infection 0.66 Cases: ‐ Antigen LFDs 740 ‐ Control (no testing) 657
[34]	RT‐PCR (pooled saliva swab “Lolli method”)	RT‐PCR (pooled buccal or oropharyngeal swab)	SARS‐CoV‐2 positivity rate: ‐ Primary school Children, pooled saliva swab 1% ‐ Primary school children, pooled buccal or oropharyngeal swab 0.4% ‐ Secondary school children, pooled saliva swab 1.3% ‐ Secondary school children, pooled buccal or oropharyngeal swab 1.3% ‐ Staff at schools, pooled buccal or oropharyngeal swab 0.5% Cases: ‐ Primary school Children, pooled saliva swab 5 ‐ Primary school children, pooled buccal or oropharyngeal swab 2 ‐ Secondary school children, pooled saliva swab 15 ‐ Secondary school children, pooled buccal or oropharyngeal swab 14 ‐ Staff at schools, pooled buccal or oropharyngeal swab 3
[36]	RT‐PCR [Cobas Liat SARS‐CoV‐2 and Influenza A/B (intervention period)]	RT‐PCR [Cobas SARS‐CoV‐2 test on the cobas 6800 System or on‐demand urgent testing using the GenMark Dx® ePlex® SARS‐CoV‐2 Test (control period)]	SARS‐CoV‐2 positivity rate: ‐ RT‐PCR (Cobas Liat SARS‐CoV‐2 and Influenza A/B) 14.05% ‐ RT‐PCR (Cobas SARS‐CoV‐2 test on the cobas 6800 System) 12.4% Cases: ‐ RT‐PCR (Cobas Liat SARS‐CoV‐2 and Influenza A/B) 638 ‐ RT‐PCR (Cobas SARS‐CoV‐2 test on the cobas 6800 System) 538 Hospitalization: ‐ RT‐PCR (Cobas Liat SARS‐CoV‐2 and Influenza A/B) 38.33% ‐ RT‐PCR (Cobas SARS‐CoV‐2 test on the cobas 6800 System) 36.32% ICU admission rate: ‐ RT‐PCR (Cobas Liat SARS‐CoV‐2 and Influenza A/B) 11.78% ‐ RT‐PCR (Cobas SARS‐CoV‐2 test on the cobas 6800 System) 14.22% Death: ‐ RT‐PCR (Cobas Liat SARS‐CoV‐2 and Influenza A/B) 0.16% ‐ RT‐PCR (Cobas SARS‐CoV‐2 test on the cobas 6800 System) 0.16%
[31]	RT‐PCR (saliva specimen)	RT‐PCR (NP swab specimen)	SARS‐CoV‐2 positivity rate: RT‐PCR (saliva specimen) ‐ Community asymptomatic group 1.07% ‐ Community all‐comers group 9.72% RT‐PCR (NP swab specimen) **‐** Hospital asymptomatic preoperative group 1.12% ‐ Hospital all‐comers group 10.83%
[33]	RT‐PCR	Antigen‐detecting RDT (BinaxNOW)	Cases: ‐ RT‐PCR 226 ‐ Antigen‐detecting RDT 127
[35]	RT‐PCR	1. POC rapid SARS CoV‐2 2. POC testing with VitaPCR	SARS‐CoV‐2 positivity rate: ‐ RT‐PCR 20.7% ‐ POC rapid SARS CoV‐2 33.2% ‐ POC testing with VitaPCR 41.5% Cases: ‐ RT‐PCR 53 ‐ POC rapid SARS CoV‐2 146 ‐ POC testing with VitaPCR 229 Hospitalization: ‐ RT‐PCR 11.6% ‐ POC rapid SARS CoV‐2 17.6% ‐ POC testing with VitaPCR 23% ICU admission rate: ‐ RT‐PCR 2.6% ‐ POC rapid SARS CoV‐2 1.4% ‐ POC testing with VitaPCR 1% Death: ‐ RT‐PCR 5.6% ‐ POC rapid SARS CoV‐2 6.2% ‐ POC testing with VitaPCR 8.5%
[42]	RT‐PCR	The lateral flow immunoassay (Panbio COVID‐19 Ag test; Abbott Laboratories)	SARS‐CoV‐2 positivity rate: ‐ The prevalence of SARS‐CoV‐2‐positive PCR results in asymptomatic participants was 0.30% ‐ SARS‐CoV‐2 prevalence in asymptomatic exposed group was 0.7% ‐ Prevalence in symptomatic students and staff was 5.1%
[43]	Point‐of‐care antigen lateral flow test (high POC‐LFT use)	1. POC‐LFT (medium POC‐LFT use) 2. POC‐LFT (low POC‐LFT use)	SARS‐CoV‐2 positivity rate: ‐ The increase of PCR positivity (from October to November) and the decline of PCR positivity (from November to December) was slightly higher in the high POC‐LFT group compared to the medium group (4.9% greater increase and 2.2% greater decrease) ‐ The increase of PCR positivity (from October to November) and the decline of PCR positivity (from November to December) was much higher in the high POC‐LFT group compared to the low group (48.8% greater increase and11.2% greater decrease) Hospitalization: The increase in hospitalization (from October to November) and the decline in hospitalization (from November to December) was slightly higher in the high POC‐LFT group compared to the medium group (4.9% greater increase and 7.9% greater decrease) ‐ The increase in PCR positivity (from October to November) and the decline of PCR positivity (from November to December) was much higher in the high POC‐LFT group compared to the low group (48.8% greater increase and 7.9% greater decrease)
[38]	RT‐PCR (clinic based)	RT‐PCR (community based)	SARS‐CoV‐2 positivity rate: ‐ RT‐PCR (clinic based) 3.2% ‐ RT‐PCR (community based) 7.1%
[39]	SD Biosensor SARS‐CoV‐2 antigen‐detecting RDT (distributed by Roche)	RT‐PCR	SARS‐CoV‐2 positivity rate: Antigen‐detecting RDT 6.1%
[40]	RT‐PCR (self‐collected nasal swabs)	RT‐PCR (healthcare worker collected)	SARS‐CoV‐2 positivity rate: ‐ RT‐PCR (self‐collected nasal swabs) 44.4%; ‐ RT‐PCR (healthcare worker collected) 44.4% Cases: ‐ RT‐PCR (self‐collected nasal swabs) 87; ‐ RT‐PCR (healthcare worker collected) 87
[44]	RT‐PCR	Antigen‐detecting RDT—SD Biosensor antigen detection test (South Korea), namely, the STANDARD F COVID‐19 Ag FIA	SARS‐CoV‐2 positivity rate: ‐ RT‐PCR 0.9% ‐ Antigen‐detecting RDT 3.2% Cases: ‐ RT‐PCR 4 ‐ Antigen‐detecting RDT 37
[30]	RT‐PCR (traditional pharyngeal swabbing method)	RT‐PCR (OPAD)	SARS‐CoV‐2 positivity rate: ‐ RT‐PCR (traditional) 45.45% ‐ RT‐PCR OPAD 54.55% Cases: ‐ RT‐PCR (traditional) 10 ‐ RT‐PCR OPAD 12
[37]	RT‐PCR (automated, high‐throughput MDx platform)	1. RT‐PCR (Cobas 6800® SARS‐CoV‐2) 2. RT‐PCR (GeneXpert® SARS‐CoV‐2 test) 3. RT‐PCR (VIASURE SARS‐CoV‐2 Real‐Time PCR Detection Kit (BD MAX™) 4. RT‐PCR (Cobas® Liat® SARS‐CoV‐2 and Influenza A/B)	Cost (reagent costs per test in Swiss francs (CHF)): ‐ RT‐PCR (MDx platform) 21 ‐ RT‐PCR (Cobas 6800® SARS‐CoV‐2) 25 ‐ RT‐PCR (GeneXpert® SARS‐CoV‐2 test) 45.25 ‐ RT‐PCR (VIASURE SARS‐CoV‐2 Real‐Time PCR Detection Kit for BD MAX™) 53 ‐ RT‐PCR (Cobas® Liat® SARS‐CoV‐2 and Influenza A/B) 42
[45]	SARS‐CoV‐2 Ag‐RDTs for dedicated staff	SARS‐CoV‐2 Ag‐RDTs for nondedicated staff	Cost: ‐ SARS‐CoV‐2 Ag‐RDTs for hospital dedicated staff: €30.12 ‐ SARS‐CoV‐2 Ag‐RDTs for hospital nondedicated: €14.56 ‐ SARS‐CoV‐2 Ag‐RDTs for Charité nondedicated: €15.03
[29]	RT‐PCR	Antigen‐detecting RDT (Standard Q COVID‐19 Ag test; (SD BIOSENSOR)	SARS‐CoV‐2 positivity rate: ‐ RT‐PCR 4.6% ‐ Antigen‐detecting RDT 3.7% Cases: ‐ RT‐PCR 221 ‐ Antigen‐detecting RDT 177 Cost: At 100,000 daily tests, cost per positive sample: ‐ RT‐PCR 236.06 USD ‐ Antigen‐detecting RDT 176.84 USD

Abbreviations: Ag‐RDT, antigen‐based rapid detection tests; COVID‐19, coronavirus disease‐19; FIA, fluorescent immunoassay; LFD, lateral flow device; MDx, molecular diagnostic; OPAD, optimized pharyngeal swab assisted device; POC‐LFT, point‐of‐care antigen lateral flow test; RDT, rapid diagnostic test; RT‐PCR, reverse transcription‐polymerase chain reaction; SARS‐CoV‐2, severe acute respiratory syndrome‐coronavirus 2.

An interactive map of the available evidence was created using the evidence for policy and practice mapper [46]. This interactive map allows for a visual exploration of the data and facilitates a better understanding of the distribution of the studies. The interactive map is available at the OSF (open science framework) project associated with this review. By utilizing the interactive map, users can navigate through the evidence and explore the studies based on different parameters, such as location, study design, and outcomes. This interactive tool enhances the accessibility and usability of the evidence presented in this review, enabling users to interact with the data and gain insights more effectively.

## 4 DISCUSSIONS

Our scoping review aimed to map the available evidence on the effectiveness of SARS‐CoV‐2 testing strategies for suspected cases and asymptomatic populations. We included 17 studies in our mapping, while highlighting an additional 40 mathematical modelling studies, which, although notable, fell outside the scope of our evidence mapping.

Regarding the outcomes, we intended to report on cases avoided, COVID‐19 positivity rate, hospitalizations, deaths, and costs. However, we did not find any data on cases avoided. Nonetheless, the included studies reported on the COVID‐19 positivity rate, and as a proxy outcome, we also noted the number of cases reported. Only three studies provided information on hospitalizations, ICU admission rates, and deaths. Additionally, three studies examined the cost of testing.

In terms of the population studied, most included studies focused on symptomatic individuals or a combination of symptomatic and asymptomatic individuals. We found fewer studies that specifically considered asymptomatic individuals or asymptomatic contacts. Moreover, there were limited studies that included healthcare workers, with only one study considering healthcare workers in addition to other participants.

In examining vaccination status, we identified only two studies that considered both vaccinated and unvaccinated populations. These studies specifically mentioned the vaccination status of the participants.

We also examined the settings of the studies. Four were conducted in educational environments, while we were unable to identify any research carried out within long‐term care facilities. Nonetheless, we did locate five studies undertaken at a community level.

In our review, we utilized the PRO‐EDI tool to explore equity, diversity, and inclusion issues. Our assessment revealed that most of the included studies only provided information on the age and sex of the study population. Notably, we did not find any reporting on gender. The ethnicity of the participants was mentioned in only seven studies. The participant's socioeconomic status, level of education, and location (urban or rural) were reported less frequently. Our findings underscore a substantial deficiency in the representation of equity, diversity, and inclusion characteristics within the studies we reviewed. Acknowledging the importance of these aspects in health research is critical, as corroborated by existing literature [47]. However, our assessment highlights the lack of the implementation of such practices in reporting these components in the included studies. The PRO‐EDI tool considers factors such as age, sex, gender, ethnicity, socioeconomic status, level of education, location, and so forth. These factors are important in the context of health disparities related to COVID‐19. Health inequalities were prominently observed during the COVID‐19 pandemic. Subgroup analyses focusing on testing, and health outcomes as per the components of PRO‐EDI could have illuminated the health disparities. However, poor reporting of equity and diversity issues in the primary research highlights the reporting gaps.

It is important to emphasize the importance of incorporating equity, diversity, and inclusion considerations in research, as it enables a comprehensive understanding of the impacts and outcomes of interventions across diverse populations. By addressing these gaps in reporting, researchers can contribute to a more inclusive and equitable evidence base that accounts for the diverse needs and experiences of different population groups.

Our evidence mapping revealed a significant gap in the studies conducted in long‐term care facilities. This contrasts with a Cochrane rapid review specifically focused on studies conducted in the setting of long‐term care facilities to prevent SARS‐CoV‐2 infections [48]. However, it is important to note that the objective of this review differed from our own. The authors of the Cochrane review sought to explore nonpharmacological measures to prevent COVID‐19 infection in long‐term care facilities.

The Cochrane review identified 11 observational studies and 11 mathematical modelling studies. While one of the included observational studies focused on routine testing of residents and staff as a preventive measure, it did not meet our inclusion criteria as our focus was specifically on comparing different testing strategies. Nonetheless, the scarcity of evidence based on studies conducted in long‐term care facilities is prominently evident.

This highlights the need for more research addressing the effectiveness of testing strategies in long‐term care facilities. Given the unique challenges and vulnerabilities in such settings, it is crucial to generate evidence to inform targeted and effective testing strategies that can help prevent SARS‐CoV‐2 infections among residents and staff in long‐term care facilities.

We also observed a limited number of studies conducted in school settings. Another relevant Cochrane review focused on preventive measures implemented in school settings [49]. This review included a total of 38 studies, out of which 33 were mathematical modelling studies. However, only one observational study reported outcomes related to the impact of mass testing on case detection.

It is important to note that this study, which reported outcomes related to the impact of mass testing, was not included in our scoping review due to the absence of a comparison group. This finding further highlights the lack of available evidence on the effectiveness of testing strategies specifically in school settings. The scarcity of studies in school settings emphasizes the need for more research to evaluate and understand the effectiveness of testing strategies in preventing and controlling SARS‐CoV‐2 infections within educational settings. Robust evidence is crucial to inform evidence‐based policies and interventions that can effectively protect students, teachers, and staff in school settings and mitigate the transmission of the virus.

Our evidence mapping identified 10 studies that used antigen‐detecting RDT and reported outcomes related to COVID‐19 positivity rate, hospitalizations, deaths, and costs.

A recent rapid review focused specifically on the effectiveness of antigen‐detecting RDT in reducing transmission among asymptomatic individuals [18]. The included studies in that review assessed the efficacy of antigen‐detecting RDT in various scenarios, such as population‐level screening, screening before specific events, and serial testing in different settings including schools and care homes.

However, it is important to note that none of the studies included in the rapid review were considered in our scoping review. This is because our scoping review specifically aimed to compare different testing strategies, and the studies in the rapid review did not meet our inclusion criteria regarding the comparability of the testing strategies.

Despite the growing body of evidence on the effectiveness of antigen‐detecting RDT, the rapid review [18] noted inconsistent findings and highlighted the low certainty of evidence. This emphasizes the need for further research and more robust studies to better understand the role of antigen‐detecting RDT in reducing transmission among asymptomatic individuals.

The wide variation in sample sizes identified in both our scoping review and the rapid review underscores the need for larger scale studies with rigorous methodologies to provide more reliable and generalizable evidence on the effectiveness of testing strategies, including the use of antigen‐detecting RDT, in different settings and populations.

## 5 RESEARCH IMPLICATION

We have identified potential scope for further synthesis. Given the variety of testing strategies (antigen‐detecting RDT, RT‐PCR) used across the studies, a further synthesis could compare the effectiveness of these strategies in terms of COVID‐19 positivity rate, number of cases, hospitalizations, ICU admissions, and deaths. However, evidence for hospitalizations, ICU admissions, and deaths is minimal. Some studies tested symptomatic and asymptomatic individuals, while others focused on one or the other. A synthesis could examine how the presence or absence of symptoms affects the outcomes of the testing strategies. Some studies focused on specific populations, such as school students or asymptomatic contacts. A synthesis could examine the effectiveness of testing strategies in these populations. We also propose a qualitative evidence synthesis on barriers and enablers to COVID‐19 testing strategies. This would provide important insights into the human and systemic factors influencing the efficacy of testing strategies across different global contexts. This knowledge is crucial for enhancing testing protocols, increasing public compliance, and ultimately controlling the spread of any future pandemic.

Future primary studies including retrospective analysis of any existing data, might focus on specific populations, such as healthcare workers, and settings such as academic institutions, and long‐term care facilities. Other considerations might include the vaccination status of the participants. Prospective studies should consider equity, diversity, and inclusion while presenting the outcome.

This scoping review was designed to inform the development of WHO recommendations for SARS‐CoV‐2 testing strategies. The findings and identified gaps will provide the basis for further exploration of synthesis on specific populations such as asymptomatic populations, asymptomatic contacts, and school students. The findings of the scoping review provide a clear picture of the existing evidence on different testing methods in terms of SARS‐CoV‐2 positivity rate, number of cases, hospitalization rate, ICU admission rate, death rate, and cost.

### 5.1 Strength and limitations

The strength of this scoping review lies in its adherence to standard guidelines and rigorous methodology. The comprehensive and broad search strategy, the duplicate screening process, and the cross‐checking of extracted data by a second reviewer enhance the reliability and validity of the review. Developing an interactive evidence map provides a visual representation of the evidence and highlights gaps in knowledge.

However, it is important to acknowledge the limitations of this scoping review. By focusing only on articles published in English, there is a possibility that relevant articles published in other languages were excluded. The lack of an in‐depth analysis and synthesis approach limits the ability to draw definitive conclusions on the effectiveness of testing methods or strategies. Additionally, the exclusion of mathematical modelling studies from the evidence mapping is another limitation of the scoping review. However, we have provided a list of our identified mathematical modelling studies.

Despite these limitations, this scoping review serves as a valuable resource by mapping the available evidence, providing insights into the characteristics of the included studies, and identifying gaps and areas for further research. It provides a foundation for future systematic reviews or primary research to explore the effectiveness of different testing methods and strategies for SARS‐CoV‐2 in diverse populations and settings.

## 6 CONCLUSION

This scoping review has successfully identified evidence gaps in the effectiveness of SARS‐CoV‐2 testing strategies, particularly in specific settings such as schools and long‐term care facilities. The identification of these gaps highlights the need for further research in these areas to generate evidence that can inform decision‐making and policy development.

Furthermore, this review has also identified gaps in reporting outcomes related to cases avoided in the community, hospitalization, death, and cost estimation. These gaps indicate areas where more comprehensive and standardized reporting is necessary to assess testing strategies' true impact and cost‐effectiveness. By identifying these gaps, this scoping review can serve as a valuable resource for researchers and policymakers, helping to prioritize and guide future research efforts in these specific areas.

Overall, this scoping review provides a foundation for further research, allowing researchers and stakeholders to focus on addressing the identified evidence gaps and improving the understanding of the effectiveness of SARS‐CoV‐2 testing strategies.

## AUTHOR CONTRIBUTIONS

**KM Saif‐Ur‐Rahman**: Conceptualization; data curation; formal analysis; funding acquisition; investigation; methodology; project administration; supervision; validation; visualization; writing—original draft; writing—review and editing. **Ani Movsisyan**: Conceptualization; formal analysis; investigation; methodology; validation; writing—review and editing. **Kavita Kothari**: Data curation; methodology; resources; software; writing—review and editing. **Thomas Conway**: Data curation; investigation; writing—review and editing. **Marie Tierney**: Data curation; investigation; writing—review and editing. **Caoimhe Madden**: Data curation; investigation; writing—review and editing. **Petek Eylul Taneri**: Data curation; investigation; writing—review and editing. **Jane A. O'Halloran**: Data curation; investigation; writing—review and editing. **Nadra Nurdin**: Data curation; investigation; writing—original draft; writing—review and editing. **Lena Murphy**: Data curation; investigation; writing—original draft; writing—review and editing. **Deirdre Mulholland**: Data curation; investigation; writing—review and editing. **Andrea C. Tricco**: Investigation; methodology; supervision; validation; writing—review and editing. **Declan Devane**: Conceptualization; formal analysis; funding acquisition; investigation; methodology; project administration; resources; software; supervision; validation; writing—review and editing.

## CONFLICT OF INTEREST STATEMENT

The authors declare no conflict of interest.

## PEER REVIEW

The peer review history for this article is available at https://www.webofscience.com/api/gateway/wos/peer-review/10.1002/cesm.12030.

## Supporting information

Supporting information.

## Data Availability

Data are available from the corresponding author upon considerable request.
