# Supplementary material for: Effectiveness of SARS‐CoV‐2 testing strategies: A scoping review
Source: Cochrane Evid Synth Methods. 2023 Nov 21;1(9):e12030. doi: 10.1002/cesm.12030 (PMC11795984; doi:10.1002/cesm.12030)
Supplement: Supplementary file 1 — Supporting information. [file CESM-1-e12030-s001.docx]

**Supplementary Table 1**: Comprehensive search strategy for Medline (OVID)

| Database: Medline (OVID) | | |
| --- | --- | --- |
| URL: www.ovidsb.com Ovid MEDLINE(R) <1946 to January Week 2 2023> | | |
| Date Searched: 24.01.2022 | | |
| # | Searches | Results |
| 1 | exp COVID-19 Testing/ | 10223 |
| 2 | (PCR OR polymerase-chain-reaction OR qPCR OR ddPCR OR reverse-transcriptase OR ribonucleic-acid OR nucleic-acid OR NAAT* OR LAMP OR loop-mediated OR isothermal-amplification OR immunoassay* OR In-vitro-diagnostic* OR IVD*) ADJ7 (test OR tests OR testing OR tested OR diagnos* OR screen* OR infection-status OR positive-result* OR positive-patient*) | 94750 |
| 3 | (antigen OR rapid OR lateral OR swab*) ADJ5 (test OR tests OR testing OR tested OR detect* OR diagnos* OR screen*) | 138974 |
| 4 | ((rapid OR lateral) ADJ3 (antigen).ti,ab,kw,kf) | 2400 |
| 5 | (antigen OR lateral-flow) ADJ3 (point-of-care OR POCT* OR RDT OR bedside-test*) | 390 |
| 6 | exp COVID-19/ OR exp SARS-CoV-2/ OR (covid* or coronavirus* or corona* virus* or Cov or "SARS-CoV-2*" or "SARSCoV-2*" or "SARSCoV2*" or "SARS-CoV2*" or "severe acute respiratory syndrome*" or Ncov* or "n-cov").ti,ab,kw,kf | 229520 |
| 7 | 1 or 2 or 3 or 4 or 5 | 224968 |
| 8 | 6 AND 7 | 18553 |
| 9 | (letter or historical article or comment or editorial or news or case reports).pt. | 4369132 |
| 10 | 8 NOT 9 | 15551 |
| 11 | limit 10 to yr="2020-Current" | 14607 |
| 12 | (animals/ NOT (animals/ AND humans/)) | 5047666 |
| 13 | 11 NOT 12 | 14454 |
| 14 | antibody.ti OR serological.ti OR seroprevelance.ti | 141909 |
| 15 | 13 NOT 14 | 13649 |

**Supplementary Table 2**: Comprehensive search strategy for Embase (elsevier)

| Database: Embase (elsevier) | | |
| --- | --- | --- |
| URL: www.embase.com | | |
| Date Searched: 24.01.2023 | | |
| # | Searches | Results |
| 1 | COVID-19 testing'/exp | 8526 |
| 2 | (PCR OR polymerase-chain-reaction OR qPCR OR ddPCR OR reverse-transcriptase OR ribonucleic-acid OR nucleic-acid OR NAAT* OR LAMP OR loop-mediated OR isothermal-amplification OR immunoassay* OR In-vitro-diagnostic* OR IVD*) NEAR/7 (test OR tests OR testing OR tested OR diagnos* OR screen* OR infection-status OR positive-result* OR positive-patient*) | 143496 |
| 3 | (antigen OR rapid OR lateral OR swab*) NEAR/5 (test OR tests OR testing OR tested OR detect* OR diagnos* OR screen*) | 222252 |
| 4 | ((rapid OR lateral) NEAR/3 (antigen)) | 3979 |
| 5 | (antigen OR lateral-flow) NEAR/3 (point-of-care OR POCT* OR RDT OR bedside-test*) | 604 |
| 6 | ('severe acute respiratory syndrome coronavirus 2'/exp OR 'coronavirus  disease 2019'/exp OR 'experimental coronavirus disease 2019'/exp OR 'covid19'/exp) OR (sarscov2 OR sars-cov OR sarscov 2 OR sars-virus OR sars2 OR sars 2 OR novel-cov OR ncov OR ncovid OR 2019ncov OR ncov19 OR coronavir* OR corona-vir* OR coronvirus OR corona-pandemic OR covid OR covid*2 OR covid19 OR covid2019):ti,ab,kw,de | 402602 |
| 7 | #1 OR #2 OR #3 OR #4 OR #5 | 349145 |
| 8 | #6 AND #7 | 29110 |
| 9 | [letter]/lim OR [conference abstract]/lim OR [conference paper]/lim OR [conference review]/lim OR [editorial]/lim OR [note]/lim | 8430117 |
| 10 | #8 NOT #9 | 21691 |
| 11 | [2020-2023]/py | 5574447 |
| 12 | #10 AND #11 | 20349 |
| 13 | ([animals]/lim NOT ([animals]/lim AND [humans]/lim)) | 6271668 |
| 14 | #12 NOT #13 | 20055 |
| 15 | antibody:ti OR serological:ti OR seroprevelance:ti | 187378 |
| 16 | #14 NOT #15 | 19085 |

**Supplementary Table 3**: Comprehensive search strategy for Europe PMC

| Database: Europe PMC | | |
| --- | --- | --- |
| URL: https://europepmc.org/ | | |
| Date Searched: 24.01.2022 | | |
| # | Searches | Results |
| 1 | (Title:"COVID-19 Testing" OR Abstract:"COVID-19 Testing" OR Title:"Sars-cov-2 Testing" OR Abstract:"Sars-cov-2 Testing") OR (Title:(PCR OR polymerase-chain-reaction OR qPCR OR ddPCR OR reverse-transcriptase OR ribonucleic-acid OR nucleic-acid OR NAAT* OR LAMP OR loop-mediated OR isothermal-amplification OR immunoassay* OR In-vitro-diagnostic* OR IVD*) AND Title:(test OR tests OR testing OR tested OR diagnos* OR screen* OR infection-status OR positive-result* OR positive-patient*)) OR (Title:(antigen OR rapid OR lateral OR swab*) AND title:(test OR tests OR testing OR tested OR detect* OR diagnos* OR screen*)) OR (Title:(rapid OR lateral) AND Title:(antigen)) OR (Title:(antigen OR lateral-flow) AND Title:(point-of-care OR POCT* OR RDT OR bedside-test*)) | 76,313 |
| 2 | ((Title:(corona virus) OR Title:(corona pandemic) OR Title:(coronavir*) OR Title:(coronavirus) OR Title:(covid19) OR Title:(covid) OR Title:(wuhan virus) OR Title:(severe acute respiratory syndrome) OR Title:(novel cov) OR Title:(ncov*) OR Title:(2019ncov) OR Title:(ncov19) OR Title:(ncovid) OR Title:(sarscov2) OR Title:(sars2) OR Title:(sars 2) OR Title:(sars-cov) OR Title:(sarscov 2) OR Title:(sarscov-2) OR Title:(sars co v 2) OR Title:(sarscov) OR Title:(sars virus) OR Title:(covid*2) OR Title:(covid2*) OR Title:(covid 19) OR Title:(covid2019) OR Title:(sars virus)) OR (Abstract:(corona virus) OR Abstract:(corona pandemic) OR Abstract:(coronavir*) OR Abstract:(coronavirus) OR Abstract:(covid19) OR Abstract:(covid) OR Abstract:(wuhan virus) OR Abstract:(severe acute respiratory syndrome) OR Abstract:(novel cov) OR Abstract:(ncov*) OR Abstract:(2019ncov) OR Abstract:(ncov19) OR Abstract:(ncovid) OR Abstract:(sarscov2) OR Abstract:(sars2) OR Abstract:(sars 2) OR Abstract:(sars-cov) OR Abstract:(sarscov 2) OR Abstract:(sarscov-2) OR Abstract:(sars co v 2) OR Abstract:(sarscov) OR Abstract:(sars virus) OR Abstract:(covid*2) OR Abstract:(covid 19) OR Abstract:(covid2019) OR Abstract:(sars virus))) | 439,008 |
| 1 AND 2 | ((Title:"COVID-19 Testing" OR Abstract:"COVID-19 Testing" OR Title:"Sars-cov-2 Testing" OR Abstract:"Sars-cov-2 Testing") OR (Title:(PCR OR polymerase-chain-reaction OR qPCR OR ddPCR OR reverse-transcriptase OR ribonucleic-acid OR nucleic-acid OR NAAT* OR LAMP OR loop-mediated OR isothermal-amplification OR immunoassay* OR In-vitro-diagnostic* OR IVD*) AND Title:(test OR tests OR testing OR tested OR diagnos* OR screen* OR infection-status OR positive-result* OR positive-patient*)) OR (Title:(antigen OR rapid OR lateral OR swab*) AND title:(test OR tests OR testing OR tested OR detect* OR diagnos* OR screen*)) OR (Title:(rapid OR lateral) AND Title:(antigen)) OR (Title:(antigen OR lateral-flow) AND Title:(point-of-care OR POCT* OR RDT OR bedside-test*))) AND ((Title:(corona virus) OR Title:(corona pandemic) OR Title:(coronavir*) OR Title:(coronavirus) OR Title:(covid19) OR Title:(covid) OR Title:(wuhan virus) OR Title:(severe acute respiratory syndrome) OR Title:(novel cov) OR Title:(ncov*) OR Title:(2019ncov) OR Title:(ncov19) OR Title:(ncovid) OR Title:(sarscov2) OR Title:(sars2) OR Title:(sars 2) OR Title:(sars-cov) OR Title:(sarscov 2) OR Title:(sarscov-2) OR Title:(sars co v 2) OR Title:(sarscov) OR Title:(sars virus) OR Title:(covid*2) OR Title:(covid2*) OR Title:(covid 19) OR Title:(covid2019) OR Title:(sars virus)) OR (Abstract:(corona virus) OR Abstract:(corona pandemic) OR Abstract:(coronavir*) OR Abstract:(coronavirus) OR Abstract:(covid19) OR Abstract:(covid) OR Abstract:(wuhan virus) OR Abstract:(severe acute respiratory syndrome) OR Abstract:(novel cov) OR Abstract:(ncov*) OR Abstract:(2019ncov) OR Abstract:(ncov19) OR Abstract:(ncovid) OR Abstract:(sarscov2) OR Abstract:(sars2) OR Abstract:(sars 2) OR Abstract:(sars-cov) OR Abstract:(sarscov 2) OR Abstract:(sarscov-2) OR Abstract:(sars co v 2) OR Abstract:(sarscov) OR Abstract:(sars virus) OR Abstract:(covid*2) OR Abstract:(covid 19) OR Abstract:(covid2019) OR Abstract:(sars virus))) | 8,186 |
| 4 | #3 AND (SRC:PPR) | 1,817 |
| 5 | #4 AND (FIRST_PDATE:[2020 TO 2023]) | 1816 |

**Supplementary Table 4**: List of excluded articles

| **Serial no.** | **Title** | **Reason for exclusion** |
| --- | --- | --- |
| 1. | A cohort study of the effect of SARS-CoV-2 point of care rapid RT-PCR at the Emergency Department on targeted admission | Inappropriate publication type |
| 2. | A nucleic acid amplification test-based strategy does not help inform return to work for healthcare workers with COVID-19 | Inappropriate publication type |
| 3. | Clinical Impact, Costs, and Cost-Effectiveness of Expanded SARS-CoV-2 Testing in Massachusetts | Inappropriate publication type |
| 4. | A rapid and cost-effective diagnostic algorithm for the detection of SARS-CoV-2 infection in the emergency area by combining highly sensitive antigenic test and RT-PCR | Diagnostic test accuracy study |
| 5. | A Rapid Nucleic-Acid-Amplification-Test-Based, Conditional-Release-to-Work Policy for Health Care Personnel with Symptoms Consistent with COVID-19 | Diagnostic test accuracy study |
| 6. | A Real-World Comparison of SARS-CoV-2 Rapid Antigen Testing versus PCR Testing in Florida | Diagnostic test accuracy study |
| 7. | Abbott R ID NOW TM COVID-19 rapid molecular assay versus Hologic R Panther Aptima TM SARS-CoV-2 assay in nasopharyngeal specimens: results from 1-year retrospective study in an emergency department | Diagnostic test accuracy study |
| 8. | Accuracy and usability of saliva and nasal rapid antigen self-testing for detection of SARS-CoV-2 infection in the general population: a head-to-head comparison | Diagnostic test accuracy study |
| 9 | Adding saliva testing to oropharyngeal and deep nasal swab testing increases PCR detection of SARS-CoV-2 in primary care and children | Diagnostic test accuracy study |
| 10. | Agreement between diagnostic methods for SARS-CoV-2 infection in children seen at the Emergency Department of a children's hospital | Diagnostic test accuracy study |
| 11. | Antigen rapid tests, nasopharyngeal PCR and saliva PCR to detect SARS-CoV-2: a prospective comparative clinical trial | Diagnostic test accuracy study |
| 12. | Antigen Test Performance Among Children and Adults at a SARS-CoV-2 Community Testing Site | Diagnostic test accuracy study |
| 13. | Antigen vs RT-PCR Tests for Screening Quarantined Students in Florida During the COVID-19 Pandemic SARS-CoV-2 Delta Variant Surge | Diagnostic test accuracy study |
| 14. | Clinical assessment of SARS-CoV-2 antigen rapid detection compared with RT-PCR assay for emerging variants at a high-throughput community testing site in Taiwan | Diagnostic test accuracy study |
| 15. | Cohort study: the accuracy of screening methods of COVID-19 in pregnancy | Diagnostic test accuracy study |
| 16. | Combined nasal- and oropharyngeal self-swab provides equivalent performance compared to professionally collected oropharyngeal swabs in detecting SARS-CoV-2 in a real-life setting | Diagnostic test accuracy study |
| 17. | Combined throat/nasal swab sampling for SARS-CoV-2 is equivalent to nasopharyngeal sampling | Diagnostic test accuracy study |
| 18. | Comparative analysis of two molecular tests for the detection of COVID-19 in Cameroon | Diagnostic test accuracy study |
| 19. | Comparative Evaluation of Rapid Salivary RT-LAMP Assay for Screening of SARS-CoV-2 Infection | Diagnostic test accuracy study |
| 20. | Comparing Rapid Ag Test and PCR in SARS-CoV-2 Management in Rural Egypt | Diagnostic test accuracy study |
| 21. | Comparison of Severe Acute Respiratory Syndrome Coronavirus 2 Screening Using Reverse Transcriptase-Quantitative Polymerase Chain Reaction or CRISPR-Based Assays in Asymptomatic College Students | Diagnostic test accuracy study |
| 22. | Detection of SARS-CoV-2 in the community by nucleic acid amplification testing of saliva | Diagnostic test accuracy study |
| 23. | Detection of SARS-CoV-2 infection by rapid antigen test in comparison with RT-PCR in a public setting | Diagnostic test accuracy study |
| 24. | Efficacy Of PanbioTM Covid-19 Ag Rapid Test In Sars-Cov-2 Detection: Comparison With RT-PCR Test | Diagnostic test accuracy study |
| 25. | Evaluation of a novel SARS-CoV-2 rapid antigenic test diagnostic value in respiratory samples; is the reported test accuracy similar to values in the real-world? A cross-sectional study | Diagnostic test accuracy study |
| 26. | Evaluation of a rapid antigen test (Panbio TM COVID-19 Ag rapid test device) for SARS-CoV-2 detection in asymptomatic close contacts of COVID-19 patients | Diagnostic test accuracy study |
| 27. | Evaluation of SARS-CoV2 antibody Rapid Diagnostic Test kits (RDTs) and Real Time-Polymerase Chain Reaction (Rt-PCR) for COVID-19 Diagnosis in Kaduna, Nigeria | Diagnostic test accuracy study |
| 28. | Field evaluation of a rapid antigen test (Panbio TM COVID-19 Ag Rapid Test Device) for COVID-19 diagnosis in primary healthcare centres | Diagnostic test accuracy study |
| 29. | Field performance of NowCheck rapid antigen test for SARS-CoV-2 in Kisumu County, western Kenya | Diagnostic test accuracy study |
| 30. | Head-to-head comparison of nasal and nasopharyngeal sampling using SARS-CoV-2 rapid antigen testing in Lesotho | Diagnostic test accuracy study |
| 31. | Nasopharyngeal Panbio COVID-19 antigen performed at point-of-care has a high sensitivity in symptomatic and asymptomatic patients with higher risk for transmission and older age | Diagnostic test accuracy study |
| 32. | Performance Evaluation of Serial SARS-CoV-2 Rapid Antigen Testing During a Nursing Home Outbreak | Diagnostic test accuracy study |
| 33. | Performance of Oropharyngeal Swab Testing Compared With Nasopharyngeal Swab Testing for Diagnosis of Coronavirus Disease 2019-United States, January 2020-February 2020 | Diagnostic test accuracy study |
| 34. | Performance of Screening for SARS-CoV-2 using Rapid Antigen Tests to Detect Incidence of Symptomatic and Asymptomatic SARS-CoV-2 Infection: findings from the Test Us at Home prospective cohort study | Diagnostic test accuracy study |
| 35. | Point-of-care detection of SARS-CoV-2 antigen among symptomatic vs. asymptomatic persons: Testing for COVID-19 vs. infectivity | Diagnostic test accuracy study |
| 36. | Prospective evaluation of the point-of-care use of a rapid antigenic SARS-CoV-2 immunochromatographic test in a paediatric emergency department | Diagnostic test accuracy study |
| 37. | Rapid antigen test to identify COVID-19 infected patients with and without symptoms admitted to the Emergency Department | Diagnostic test accuracy study |
| 38. | Saliva is superior over nasopharyngeal swab for detecting SARS-CoV2 in COVID-19 patients | Diagnostic test accuracy study |
| 39. | Screening for SARS-CoV-2 infection in asymptomatic individuals using the Panbio COVID-19 antigen rapid test (Abbott) compared with RT-PCR: a prospective cohort study | Diagnostic test accuracy study |
| 40. | Self-Collected Oral Fluid and Nasal Swabs Demonstrate Comparable Sensitivity to Clinician Collected Nasopharyngeal Swabs for Covid-19 Detection | Diagnostic test accuracy study |
| 41. | The Challenge of Using an Antigen Test as a Screening Tool for SARS-CoV-2 Infection in an Emergency Department: Experience of a Tertiary Care Hospital in Southern Italy | Diagnostic test accuracy study |
| 42. | The comparative superiority of IgM-IgG antibody test to real-time reverse transcriptase PCR detection for SARS-CoV-2 infection diagnosis | Diagnostic test accuracy study |
| 43. | The dark side of SARS-CoV-2 rapid antigen testing: screening asymptomatic patients | Diagnostic test accuracy study |
| 44. | A statistical model of COVID-19 testing in populations: effects of sampling bias and testing errors | Inappropriate outcome |
| 45. | Acceptability of OP/Na swabbing for SARS-CoV-2: a prospective observational cohort surveillance study in Western Australian schools | Inappropriate outcome |
| 46. | Acceptance of Different Self-sampling Methods for Semiweekly SARS-CoV-2 Testing in Asymptomatic Children and Childcare Workers at German Day Care Centers: A Nonrandomized Controlled Trial | Inappropriate outcome |
| 47. | Comparing Nasopharyngeal and Midturbinate Nasal Swab Testing for the Identification of Severe Acute Respiratory Syndrome Coronavirus 2 | Inappropriate outcome |
| 48 | Comparison of nasopharyngeal and oropharyngeal swabs for SARS-CoV-2 detection in 353 patients received tests with both specimens simultaneously | Inappropriate outcome |
| 49. | Comparison of Unsupervised Home Self-collected Midnasal Swabs With Clinician-Collected Nasopharyngeal Swabs for Detection of SARS-CoV-2 Infection | Inappropriate outcome |
| 50. | Feasibility of a surveillance programme based on gargle samples and pool testing to prevent SARS-CoV-2 outbreaks in schools | Inappropriate outcome |
| 51. | Lessons from low seroprevalence of SARS-CoV-2 antibodies in schoolchildren: A cross-sectional study | Inappropriate outcome |
| 52. | SARS-CoV-2 Reverse Transcription-Polymerase Chain Reaction Positivity and Seroprevalence among Health Care Workers in a Referral Cancer Institute: A Cross-sectional Study | Inappropriate outcome |
| 53. | SARS-CoV-2 Screening Testing in Schools: A Comparison of School- Vs. Home-Based Collection Methods | Inappropriate outcome |
| 54. | Serial Laboratory Testing for SARS-CoV-2 Infection Among Incarcerated and Detained Persons in a Correctional and Detention Facility - Louisiana, April-May 2020 | Inappropriate outcome |
| 55. | Serial Testing for SARS-CoV-2 and Virus Whole Genome Sequencing Inform Infection Risk at Two Skilled Nursing Facilities with COVID-19 Outbreaks - Minnesota, April-June 2020 | Inappropriate outcome |
| 56. | Seroepidemiology of SARS-CoV-2 in healthcare personnel working at the largest tertiary COVID-19 referral hospitals in Mexico City | Inappropriate outcome |
| 57. | The COVID-19 Self-Testing through Rapid Network Distribution (C-STRAND) trial: A randomized controlled trial to increase COVID-19 testing in underserved populations | Inappropriate outcome |
| 58. | Very High Negative Concordance Rate of RT-PCR for SARS-CoV-2 in Nasopharyngeal Swab and Tracheo-Bronchial Aspirate in Children | Inappropriate outcome |

**Supplementary Table 5**: List of mathematical modelling studies

| **Serial no.** | **Author, Year** | **Title** |
| --- | --- | --- |
| 1. | Yapeng Cui et al, 2021 | A network-based model to explore the role of testing in the epidemiological control of the COVID-19 pandemic |
| 2. | Jonathan Karnon et al, 2022 | An Economic Evaluation of Government-Funded COVID-19 Testing in Australia |
| 3. | Emily A Kendall et al, 2021 | Antigen-based rapid diagnostic testing or alternatives for diagnosis of symptomatic COVID-19: A simulation-based net benefit analysis |
| 4. | Yu-Hao Zhou et al, 2021 | An Optimal Nucleic Acid Testing Strategy for COVID-19 during the Spring Festival Travel Rush in Mainland China: A Modelling Study |
| 5. | Leila F Dantas et al, 2021 | App-based symptom tracking to optimize SARS-CoV-2 testing strategy using machine learning |
| 6. | Trystan Leng et al, 2022 | Assessing the impact of lateral flow testing strategies on within-school SARS-CoV-2 transmission and absences: A modelling study |
| 7. | A David Paltiel et al, 2021 | Clinical and Economic Effects of Widespread Rapid Testing to Decrease SARS-CoV-2 Transmission |
| 8. | Anne M Neilan et al, 2021 | Clinical Impact, Costs, and Cost-effectiveness of Expanded Severe Acute Respiratory Syndrome Coronavirus 2 Testing in Massachusetts |
| 9. | Fergus J Chadwick et al, 2022 | Combining Rapid Antigen Testing and Syndromic Surveillance Improves Community-Based COVID-19 Detection in Low-to-Middle-Income Countries |
| 10. | CR Wells et al, 2021 | Comparative analyses of FDA EUA-approved rapid antigen tests and RT-PCR for COVID-19 quarantine and surveillance-based isolation |
| 11. | Zhanwei Du et al, 2021 | Comparative cost-effectiveness of SARS-CoV-2 testing strategies in the USA: a modelling study |
| 12. | Jay Love et al, 2021 | Comparison of antigen- and RT-PCR-based testing strategies for detection of Sars-Cov-2 in two high-exposure settings |
| 13. | Nicholas C Grassly et al, 2020 | Comparison of molecular testing strategies for COVID-19 control: a mathematical modelling study |
| 14. | Eun Young Kim et al, 2022 | Cost analysis of coronavirus disease 2019 test strategies using pooled reverse transcriptase-polymerase chain reaction technique |
| 15. | B Arwah et al, 2023 | Cost & Cost-Effectiveness of Implementing SD Biosensor Antigen Detecting SARs-CoV-2 Rapid Diagnostic Tests in Kenya |
| 16. | Sigal Maya et al, 2022 | Cost-effectiveness of antigen testing for ending COVID-19 isolation |
| 17. | Sigal Maya et al, 2022 | COVID-19 Testing Strategies for K-12 Schools in California: A Cost-Effectiveness Analysis |
| 18. | Joel Hellewell et al, 2021 | Estimating the effectiveness of routine asymptomatic PCR testing at different frequencies for the detection of SARS-CoV-2 infections |
| 19. | Anthony Terriau et al, 2021 | Estimating the impact of virus testing strategies on the COVID-19 case fatality rate using fixed-effects models |
| 20. | M Solís et al, 2022 | Estimating the performance of mass testing strategies for COVID-19: a case study for Costa Rica |
| 21. | Amelia Van Pelt et al, 2021 | Evaluation of COVID-19 Testing Strategies for Repopulating College and University Campuses: A Decision Tree Analysis |
| 22. | Johannes Forster et al, 2022 | Feasibility of SARS-CoV-2 Surveillance Testing Among Children and Childcare Workers at German Day Care Centers: A Nonrandomized Controlled Trial |
| 23. | Gregory D Lyng et al, 2021 | Identifying optimal COVID-19 testing strategies for schools and businesses: Balancing testing frequency, individual test technology, and cost |
| 24. | Isaac See et al, 2021 | Modeling Effectiveness of Testing Strategies to Prevent Coronavirus Disease 2019 (COVID-19) in Nursing Homes-United States, 2020 |
| 25. | Matt Stevenson et al, 2021 | Modelling of hypothetical SARS-CoV-2 point of care tests for routine testing in residential care homes: rapid cost-effectiveness analysis |
| 26. | Roland Diel et al, 2021 | Point-of-Care COVID-19 Antigen Testing in Exposed German Healthcare Workers-A Cost Model |
| 27. | CG McAloon et al, 2022 | Potential application of Rapid Antigen Diagnostic Tests for the detection of infectious individuals attending mass gatherings – a simulation study |
| 28. | Saskia Ricks et al, 2021 | Quantifying the potential value of antigen-detection rapid diagnostic tests for COVID-19: a modelling analysis |
| 29 | Phillip P Salvatore et al, 2022 | Quantitative Comparison of SARS-CoV-2 Nucleic Acid Amplification Test and Antigen Testing Algorithms: A Decision Analysis Simulation Model |
| 30. | S Girdwood et al, 2021 | Quantifying Rapid Antigen Diagnostic Resource Requirements for SARS-CoV-2: A Cost-Effectiveness Analysis of Testing Strategies for Five Countries in Sub-Saharan Africa |
| 31. | Billy J Quilty et al, 2021 | Quarantine and testing strategies in contact tracing for SARS-CoV-2: a modelling study |
| 32. | David R M Smith et al, 2022 | Rapid antigen testing as a reactive response to surges in nosocomial SARS-CoV-2 outbreak risk |
| 33. | Mathew V Kiang et al, 2021 | Routine asymptomatic testing strategies for airline travel during the COVID-19 pandemic: a simulation study |
| 34. | Y Jo et al, 2020 | Serological testing in addition to PCR screening for the re-opening of American colleges and universities: potential for cost-savings without compromising pandemic mitigation |
| 35. | Ali Asgary et al, 2021 | Simulating preventative testing of SARS-CoV-2 in schools: policy implications |
| 36. | Andreas Deckert et al, 2020 | Simulation of pooled-sample analysis strategies for COVID-19 mass testing |
| 37. | Samuel Clifford et al, 2021 | Strategies to reduce the risk of SARS-CoV-2 importation from international travellers: modelling estimations for the United Kingdom, July 2020 |
| 38. | Zafar Zafari et al, 2021 | The cost-effectiveness of common strategies for the prevention of transmission of SARS-CoV-2 in universities |
| 39. | Yawen Jiang et al, 2020 | The cost-effectiveness of conducting three versus two reverse transcription-polymerase chain reaction tests for diagnosing and discharging people with COVID-19: evidence from the epidemic in Wuhan, China |
| 40. | RH Baillargeon et al, 2021 | Using rapid (point-of-care) tests for COVID-19: A decision analysis comparing the expected benefit of two screening strategies |
